# Mycobacteria emulsified in olive oil-in-water trigger a robust immune response in bladder cancer treatment

**DOI:** 10.1038/srep27232

**Published:** 2016-06-06

**Authors:** Estela Noguera-Ortega, Núria Blanco-Cabra, Rosa Maria Rabanal, Alejandro Sánchez-Chardi, Mónica Roldán, Sandra Guallar-Garrido, Eduard Torrents, Marina Luquin, Esther Julián

**Affiliations:** 1 Departament de Genètica i de Microbiologia, Facultat de Biociències, Universitat Autònoma de Barcelona, Spain; 2 Unitat de Patologia Murina i Comparada, Departament de Medicina i Cirurgia Animals, Facultat de Veterinària, Universitat Autònoma de Barcelona, Spain; 3 Servei de Microscopia, Universitat Autònoma de Barcelona, Spain; 4 Bacterial Infections and Antimicrobial Therapy group, Institute for Bioengineering of Catalonia (IBEC), Spain.

## Abstract

The hydrophobic composition of mycobacterial cell walls leads to the formation of clumps when attempting to resuspend mycobacteria in aqueous solutions. Such aggregation may interfere in the mycobacteria-host cells interaction and, consequently, influence their antitumor effect. To improve the immunotherapeutic activity of *Mycobacterium brumae*, we designed different emulsions and demonstrated their efficacy. The best formulation was initially selected based on homogeneity and stability. Both olive oil (OO)- and mineral oil-in-water emulsions better preserved the mycobacteria viability and provided higher disaggregation rates compared to the others. But, among both emulsions, the OO emulsion increased the mycobacteria capacity to induce cytokines’ production in bladder tumor cell cultures. The OO-mycobacteria emulsion properties: less hydrophobic, lower pH, more neutralized zeta potential, and increased affinity to fibronectin than non-emulsified mycobacteria, indicated favorable conditions for reaching the bladder epithelium *in vivo*. Finally, intravesical OO-*M. brumae*-treated mice showed a significantly higher systemic immune response, together with a trend toward increased tumor-bearing mouse survival rates compared to the rest of the treated mice. The physicochemical characteristics and the induction of a robust immune response *in vitro* and *in vivo* highlight the potential of the OO emulsion as a good delivery vehicle for the mycobacterial treatment of bladder cancer.

Intravesical instillation of *Mycobacterium bovis* bacillus Calmette-Guerin (BCG) is an effective treatment routinely used for high-risk non-muscle-invasive bladder cancer (BC) and carcinoma *in situ* (CIS) patients^1^. Current evidence suggests that BCG therapy prevents recurrence and progression, which prolongs the patient’s survival^2^,^3^. Although the benefits of BCG immunotherapy are clear, the drawbacks should not be underestimated. The majority of BCG-treated patients do not suffer severe adverse events, but approximately 5% of patients suffer side effects that are considered to be serious, and BCG infection cases have been detected^4^. As part of efforts to seek safer alternatives to BCG, we recently demonstrated the antitumor efficacy of the non-pathogenic *Mycobacterium brumae. M. brumae* was initially isolated from soil and water samples in Barcelona (Spain)^5^,^6^. No infection cases have been described until now either in humans or animals^7^. Similar to BCG, *M. brumae* inhibits BC cell growth *in vitro* and *in vivo*, which triggers an antitumor profile in the immune cells^8^,^9^.

The antitumor effect of mycobacteria, however, could be theoretically improved. Mycobacteria cells possess a high content of lipids in their cell walls (over 60% of their weight), which provides them with an elevated hydrophobic character that leads cells to form clumps of variable size and shape in aqueous solutions^10^. This tendency for clumping makes it difficult to obtain stable and homogeneous mycobacteria suspensions. In fact, the BCG aggregates in the usual aqueous solutions that are used for intravesical instillation in BC patients in a time-dependent manner, which affects the antitumor activity^11^,^12^. Therefore, for an adequate interaction between mycobacteria and target cells, it appears that clumps should be avoided. Thus, obtaining homogenous mycobacteria suspensions could influence their antitumor effect.

One of the possible strategies is the formulation of oil-in-water (O/W) or water-in-oil (W/O) mycobacteria emulsions, which have been developed for different types of compounds to enhance the induced immune response of antitumor agents or adjuvants^13^. For example, an O/W preparation of mytomycin, a chemotherapy agent that is used for intravesical BC treatment, has recently been conducted to enhance its antitumor effect. The microemulsion of mytomycin provides longer stability to the drug (a longer biological half-life) and reduces its severe side effects after systemic administration^14^.

In the case of mycobacteria, researchers who have proposed the use of heat-killed mycobacteria or cell wall extracts for cancer treatment or as adjuvants have also developed formulations using oils. In fact, the well-known Complete Freund’s Adjuvant (CFA) is a heat-killed preparation of *Mycobacterium tuberculosis* H37Ra that is made by using mineral oil (MO)^15^. CFA is usually mixed with water-soluble antigens to make W/O emulsions, which enhance and prolong the immune response against a mixture of antigens^15^. Specifically, in all of these cited formulations, the used oils were mainly MO and soybean oil (SO), and squalene (SE), a natural organic lipid biochemical intermediate in sterol synthesis (Supplementary Table 1). To the best of our knowledge, only one previous article reported studying the administration of extracts from killed mycobacteria using olive oil (OO) for cancer treatment; however, no formulation was made because the mycobacteria extract was directly resuspended using OO^16^.

In the case of BC, treatment with live mycobacteria is required to obtain an optimum antitumor effect^9^,^8^. Thus, to improve the antitumor activity of live mycobacteria, we aimed to develop a formulation that can disaggregate mycobacteria clumps while ensuring the viability of the mycobacteria. We also aimed to demonstrate the ability of this formulation to trigger an proper immune response and to inhibit bladder tumor growth both *in vitro* and *in vivo*.

## Results

### O/W emulsion by the rod sonication protocol provided the most homogeneous mycobacteria suspension

**Figure 1:**
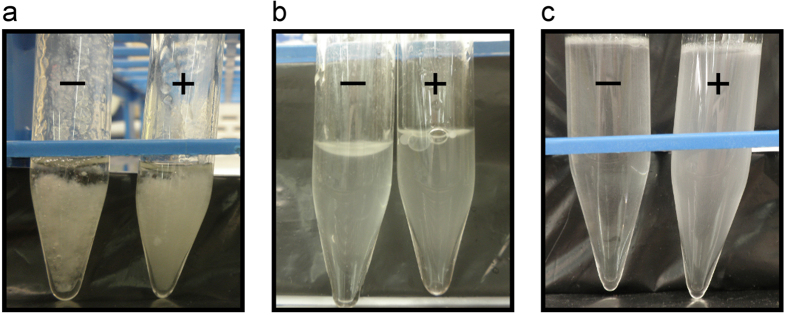
Macroscopic appearance of different OO-mycobacteria emulsions. (**a**) W/O and (**b**) O/W emulsions obtained following the sonication protocol; (c) O/W emulsion following the rod sonication protocol. Right tubes (+) correspond to *M. brumae* preparations, and left tubes (–) are without mycobacteria. OO, olive oil; W/O, water-in-oil; O/W, oil-in-water.

The initial criterion that was chosen for optimizing the mycobacteria emulsion was based on the homogeneity of the suspension and the simplicity of the protocol. Visual inspection showed that there was no homogeneity in the emulsions made using the simple sonication protocol or when the W/O emulsions were made (Fig. 1a,b). By contrast, when the O/W emulsions were made using the rod sonication protocol, homogeneous emulsions were obtained. We also observed that if the mycobacteria were blended with the oil that was already mixed with the surfactant, the emulsion was more homogeneous (Fig. 1c).

### The OO emulsion maintains the highest proportion of *M. brumae* viable cells, whereas the MO emulsion better disaggregates the *M. brumae* clumps

**Figure 2:**
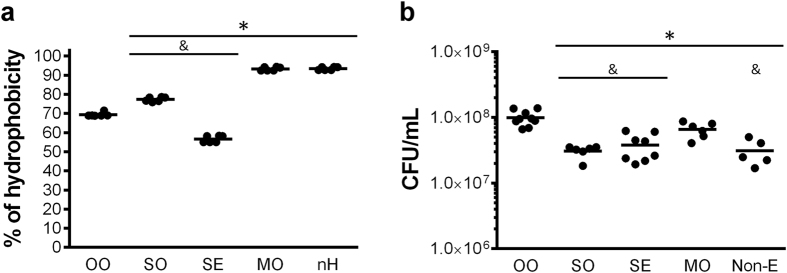
Affinity of *M. brumae* for the different tested compounds and CFU counts in each emulsion. (**a)** The hydrophobicity index was calculated from the means ± SEMs of the absorbance values from triplicate preparations of two different experiments. *p < 0.01 *versus* OO; ^&^p < 0.01 *versus* MO (Mann-Whitney *U* test). (**b)** CFU of *M. brumae* in the different emulsions or in PBS-Tween (Non-E). Values are expressed as the mean ± SEM from bacterial culture triplicates of at least three independent experiments. *p < 0.05, *versus* OO-emulsified *M. brumae*; ^&^p < 0.05 *versus* MO-emulsified *M. brumae* (Mann-Whitney *U* test). OO, olive-oil; SO, soybean oil; SE, squalene; MO, mineral oil; nH, n-hexadecane; Non-E, mycobacteria in PBS-Tween.

After selecting the protocol for making the emulsion, we evaluated the affinity of *M. brumae* for the different tested compounds. As Fig. 2a and Supplemental Fig. 1 show, *M. brumae* has a high tendency to adhere to all lipophilic compounds, indicated by elevated hydrophobicity index. The highest affinity of *M. brumae* was to MO (Fig. 2a), being similar to n-hexadecane, used as a positive control^17^.

**Figure 3:**
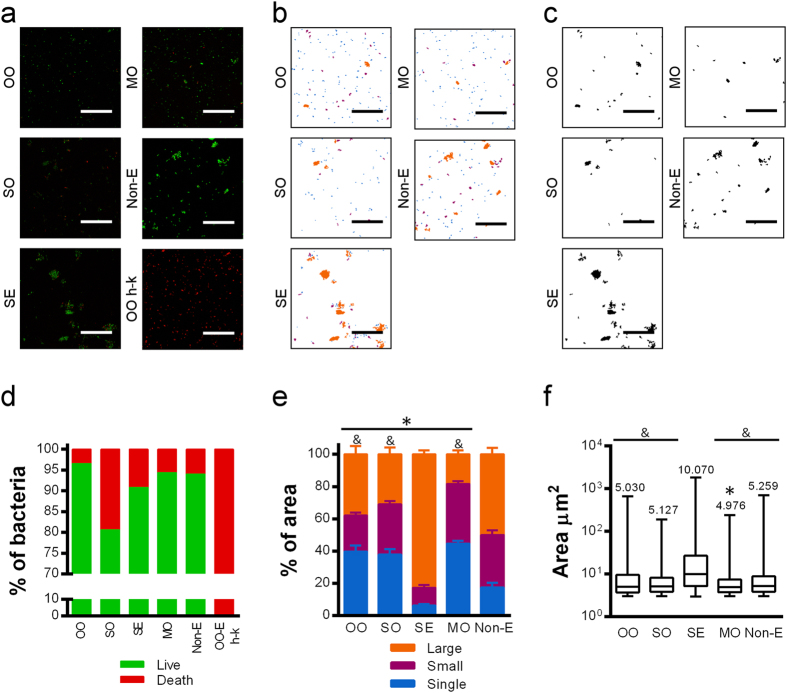
Viability and aggregation of *M. brumae* in O/W emulsions. (**a)** Representative confocal capture images of *M. brumae* emulsified using different compounds, where live bacteria are shown in green (Syto^®^ 9 staining) and dead bacteria in red (propidium iodide staining). (**b)** A binary mask representing single mycobacteria cells (in blue), small clumps (in purple) and large clumps (in orange). (**c)** A binary mask representing only aggregates. The bars indicate 40 μm. (**d)** Mean percentage of live (green columns) and dead (red columns) bacteria present in the emulsion with respect to the total counts of green and red bacteria in “**a**” confocal images. (**e)** Means ± SEMs of the percentage of the area occupied by a single bacterium with respect to the total area (in blue) and the area occupied by bacteria forming each type of aggregate (small in purple, and large in orange) obtained from the “**b**” binary mask. *p < 0.01, differences in the percentage of single cells *versus* Non-E; ^&^p < 0.001, differences in the percentage of single cells *versus* SE (Mann-Whitney *U* test). (**f)** Aggregate size determined from the “**c**” binary mask. The median size of the aggregates is indicated on top of each box. Error bars indicate range. *p < 0.01 *versus* Non-E; ^&^p < 0.0001 *versus* SE (Mann-Whitney *U* test). Values in ‘d’, ‘e’ and ‘f’ were obtained by analyzing 20 fields per condition. OO, olive-oil; SO, soybean oil; SE, squalene; MO, mineral oil, Non-E, mycobacteria in PBS-tween; OO-E h-k, olive-oil emulsion of heat-killed *M. brumae* was used as a control.

Considering that mycobacteria showed a high affinity for all of the compounds studied, we aimed to unravel how mycobacteria behave in each of them. When *M. brumae* viability was studied, we observed that OO maintains mycobacteria viability better than the rest, followed by MO emulsion (Fig. 2b, and Fig. 3a,d). By contrast, the SO emulsion diminished *M. brumae* viability to the greatest extent.

With regard to *M. brumae* clumping, MO was the oil that better disaggregated the mycobacteria clumps, obtaining smaller clumps and more isolated cells than the other oils (Fig. 3b,c,e,f). Remarkably, SE emulsified *M brumae* and made the largest clumps, as is clearly seen in the confocal images (Fig. 3c,f).

### *M. brumae* emulsified in OO triggered the highest cytokine production in infected tumor cell cultures

**Figure 4:**
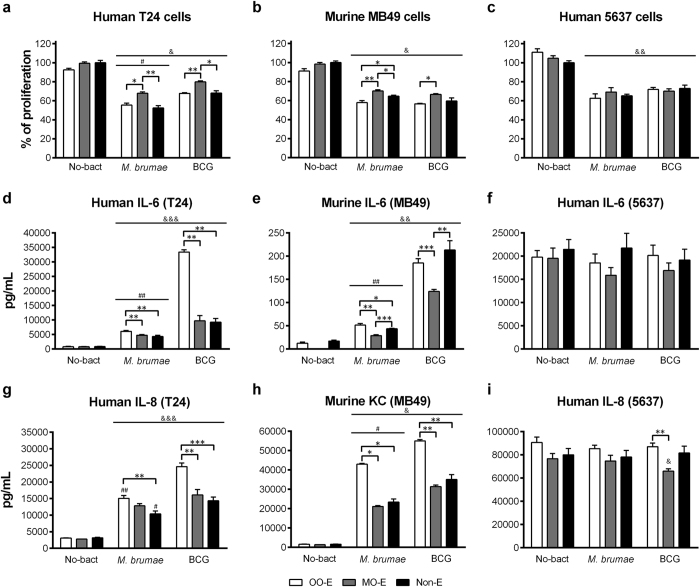
Tumor growth inhibition and cytokine production triggered by emulsified or non-emulsified mycobacteria. High grade human T24 (**a**) and murine MB49 (**b**), and human low grade 5637 (**c**) BC cells were treated with mycobacteria in OO emulsion (white columns), in MO emulsion (gray columns) or in culture media (Non-E, black columns). Emulsions without bacteria or cell culture media were added to parallel wells (No-bact). In (**a–c)** results from the MTT assay are shown as the percentage of proliferation in relation to the control cells (Non-E No-bact). Data are expressed as the means ± SEMs of the cell culture technical triplicates of three independent experiments. IL-6 (**d–f**) and IL-8/KC (**g–i**) production by infected and non-infected (No-bact) BC cells. Results are shown as the means ± SEMs of two technical replicates of the cell culture supernatants. *p < 0.05; **p < 0.01; ***p < 0.001; ^&^p < 0.05; ^&&^p < 0.01; ^&&&^p < 0.001 *versus* the respective control No-bact; ^#^p < 0.05; ^##^p < 0.01 *versus* the respective BCG (Kruskal-Wallis *H* test followed by Mann-Whitney *U* test). OO, olive oil; MO, mineral oil; Non-E, mycobacteria in PBS-tween; No-Bact, preparations without mycobacteria.

After selecting OO and MO since they were best at preserving the mycobacteria viability and disaggregating the clumps, we aimed to demonstrate *in vitro* the antitumor capacity of emulsified mycobacteria in these oils. First, a previously described infection protocol^9^,^18^ was optimized to ensure that the emulsion was completely removed from the culture after washing the cells 3 hours after infection. This goal was confirmed when we observed that the cultures treated with the emulsion without mycobacteria (considered the negative control) did not affect BC cell proliferation (Fig. 4a,b,c).

As Fig. 4 shows, OO-emulsified mycobacteria inhibited the proliferation of all three BC cell lines to a similar degree to non-emulsified mycobacteria. Only in the case of infecting BC cells with OO/W-*M. brumae,* the inhibition of MB49 growth was higher than infecting with non-emulsified mycobacteria. Remarkably, MO-emulsified mycobacteria were the least efficacious in inhibiting the BC cell proliferation in T24 and MB49 cells (p < 0.05).

Unlike the growth inhibition results, the production of cytokines by infected cells differed clearly between infection with emulsified and non-emulsified mycobacteria. As Fig. 4 shows, except for the production of IL-6 in MB49 cell culture supernatants after BCG infection, OO/W-mycobacteria triggered significantly higher levels of cytokines than the other conditions (p < 0.05). The exception was the 5736 cell line in which no differences were detected in cytokine production between infected and non-infected cultures. In fact, this cell line is characterized by elevated constitutive levels of cytokine production (Fig. 4f,i), its culture supernatant being used as a source of cytokines^19^.

### Emulsified and non-emulsified mycobacteria show similar survival rates inside BC cells

**Figure 5:**
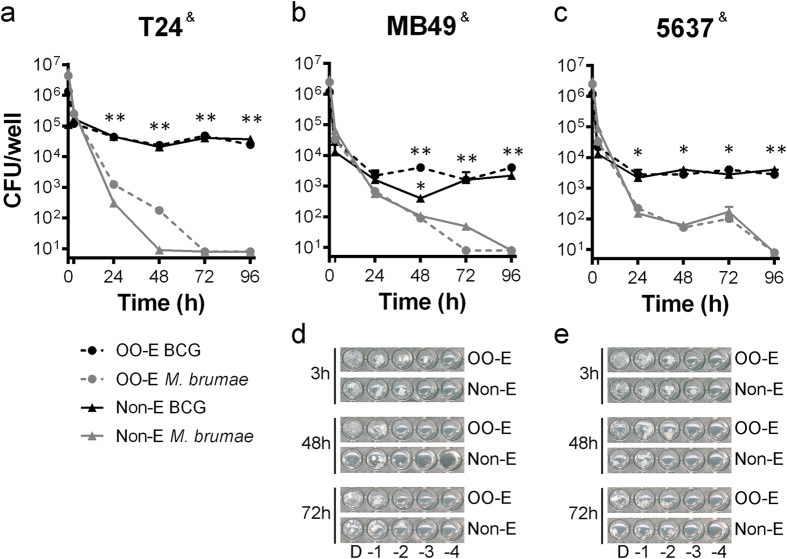
BCG and *M. brumae* survival inside T24, MB49 and 5637 BC cells. Colony-forming units (CFUs) from cell lysates at different time-points after infection are represented (h, hours). Values represent the means ± SEMs of three serial dilutions of triplicate culture wells of *M. brumae* infected T24 cells (**a**), MB49 cells (**b**) and 5637 cells (**c**), and BCG-infected T24 cells (**a**). Data are representative of one out of three (for T24) and two (for MB49 and 5637) independent experiments. BCG counts were estimated from the results obtained in the 96-well plate 7H10-based spot assay from dilutions of infected MB49 and 5637 BC cells. ^&^ < 0.001 (Friedman test); *p < 0.05; **p < 0.01 *versus* their respective *M. brumae*. (Kruskal-Wallis *H* test followed by Mann-Whitney *U* test). Representative pictures of the wells in which grown BCG from MB49 (**d**) cultures and 5637 (**e**) can be observed. OO-E, olive oil emulsion; MO-E, mineral oil emulsion; Non-E, non-emulsified preparation.

After observing *in vitro* the immunostimulatory superiority of mycobacteria formulated in the OO emulsion, we aimed to demonstrate that the formulation does not affect the non-pathogenicity of *M. brumae*. As Fig. 5 shows, *M. brumae* (both in the emulsion and in PBS) does not persist inside BC cells three-four days after infection, whereas BCG remains viable under both conditions.

### OO/W-*M. brumae* would better interact with bladder epithelium than non-emulsified-*M. brumae*

**Figure 6:**
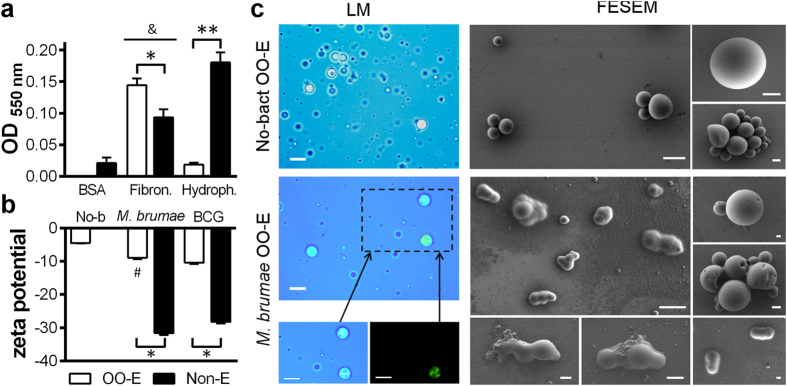
Characterization of OO-*M. brumae* emulsion. (**a)** Adhesion of *M. brumae* to BSA-coated (control), fibronectin-coated and uncoated (hydrophobicity assay) polystyrene plates. The bars represent means ± SEMs of absorbance values of triplicate wells from three independent experiments. ^&^p < 0.05 *versus* respective BSA-coated wells; *p < 0.05; **p < 0.001 (Mann-Whitney *U* test). (**b)** Zeta potential of mycobacteria preparations. The results are expressed as the mean ± SEM of 20 measurements of each triplicate sample from two independent experiments. ^#^p < 0.01 *versus* OO-E BCG; *p < 0.001 *versus* Non-E (Mann-Whitney *U* test). (**c)** Light microscopy (LM): trypan blue and fluorescence staining in upper images and separated canals in bottom images, and Field Emission Scanning Electron Microscopy (FESEM) images of OO emulsions with (*M. brumae* OO-E) or without (No-bact OO-E) *M. brumae*. Bars: 10 μm in images of LM; in FESEM: 5 μm, in the general views (2 large images), and 1 μm, in images of the details. OO, olive oil; Non-E, mycobacteria in PBS-tween; No-b, No-bact, preparation without bacteria; BSA, Bovine Serum Albumina; Fibron, fibronectin; Hydrofob, Hydrofobicity assay.

The physicochemical properties could also be crucial, *a priori*, for a favorable interaction with the bladder epithelium. Thus, different parameters were analyzed. We first observed the reduced capacity of OO/W-*M. brumae* to adhere to polystyrene plates with respect to non-emulsified *M. brumae* (Fig. 6a), which reflects the reduced hydrophobicity of the OO/W-*M. brumae*. Interestingly, OO/W-*M. brumae* bound specifically and more efficiently to fibronectin than non-emulsified *M. brumae* (p < 0.05) (Fig. 6a). OO/W-mycobacteria also showed a significantly neutralized zeta potential (Fig. 6b) with respect to non-emulsified mycobacteria. Moreover, the emulsion of mycobacteria had a pH of approximately 5 compared with 7.4 obtained when the mycobacteria were resuspended in PBS.

We also confirmed the O/W nature of the emulsion by using different techniques. When the OO/W-*M. brumae* was placed into cold water, its behavior corresponded to an O/W emulsion, in which it is completely diluted after shaking the plate (Supplementary Fig. 2). We further corroborated the nature of the emulsion by visualization using microscopy techniques (Fig. 6c). Oil droplets containing mycobacteria were clearly observed when comparing the images from light and fluorescent microscopy. Moreover, by Field Emission Scanning Electron Microscopy (FESEM), high non-uniform spheres with surface holes were observed in the OO/W-*M. brumae* emulsion compared with the small uniform spheres that were observed in the empty emulsion (No-bact) (Fig. 6c). It can be observed that in most cases, oil covered one or two mycobacterial cells (Fig. 6c), which confirms the results obtained by confocal microscopy (Fig. 3). Finally, no bacilli without oil droplets were observed in any sample.

### OO/W-mycobacteria elicits a robust immune response in tumor-bearing mice

**Figure 7:**
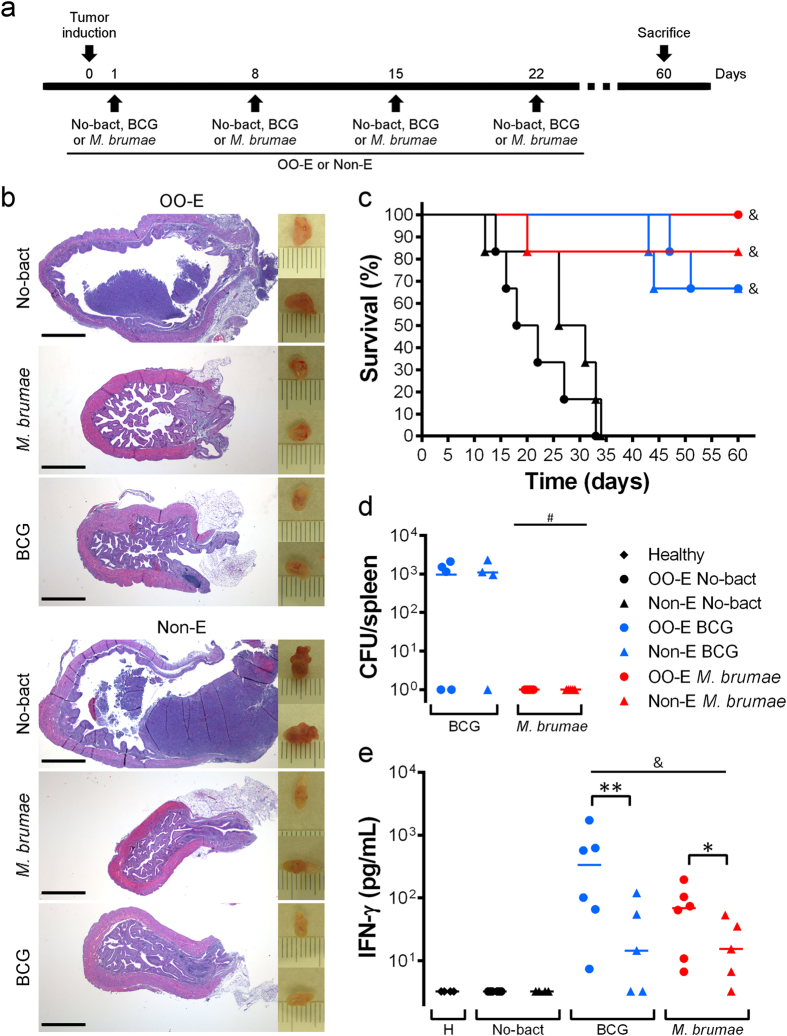
*In vivo* treatment with OO-emulsified mycobacteria. (**a)** Schematic schedule of the induction of the tumor and treatments. C57BL/6 mice (n = 6) received four weekly intravesical instillations (indicated by arrows) of only the vehicle: emulsion alone (No-bact OO-E) or PBS (No-bact Non-E) or mycobacteria in emulsion (OO-E) or in PBS (Non-E), after tumor implantation. (**b)** On the right, macroscopic pictures taken from representative bladders of tumor-bearing mice after receiving the different treatments. Each mark is 1 mm. On the left, histological images (hematoxylin-eosin staining) of bladder sections from the same bladders. Scale bar, 1 mm. (**c)** Kaplan-Meier curve of survival in tumor-bearing mice after treatments. ^&^p < 0.05 *versus* respective no mycobacteria groups (Log-Rank Mantel-Cox tests). (**d)** Colony-forming units (CFU) of splenocytes cultures from treated mice. The means of three replicates of the viable bacteria from each spleen is represented as a dot; the line identifies the mean of the animal group. ^#^p < 0.05 *versus* respective BCG groups (Mann-Whitney *U* test). (**e)** Production of IFN-γ by mycobacteria-stimulated splenocytes from treated mice measured 72 hours after stimulation. Dots represent the means of two technical replicates per spleen; the line identifies the mean of the animal group. ^&^p < 0.05 *versus* PBS group; *p < 0.05; **p < 0.01 (Mann-Whitney *U* test).

Finally, we evaluated the *in vivo* activity of OO/W-mycobacteria in the murine orthotopic model of BC. At 9 ± 2 days after tumor induction, all of the animals presented hematuria, which is a hallmark of an established tumor^9^,^20^. While 100% of the non-treated animals died within 40 days of post-tumor implantation, the mycobacteria treatment significantly prolonged survival in the tumor-bearing mice (p < 0.05). Remarkably, while 83.33% of non-emulsified *M. brumae*-treated mice survived, 100% of the tumor-bearing mice survived after OO/W-*M. brumae* treatment (Kaplan-Meier analysis) (Fig. 7c). With regard to the BCG treatments, only a slight delay in the deaths of the mice treated with OO-emulsified BCG were observed with respect to mice that were treated with BCG in PBS. There were no significant differences in the survival rates among the mycobacteria-treated groups. Interestingly, no *M. brumae* CFUs were recovered from the spleens of the treated animals. By contrast, BCG CFUs were recovered from some of the BCG-treated mice (Fig, 7d), as described previously^9^.

When the systemic immune response was analyzed, we observed that splenocyte cultures from OO/W-mycobacteria treated mice produced higher levels of IFN-γ than splenocytes from non-emulsified-mycobacteria treated mice, using either BCG or *M. brumae* as treatment (p < 0.05) (Fig. 7e). This response was specific because no cytokines were detected in supernatant from splenocyte cultures of non-mycobacteria treated tumor-bearing mice or healthy mice (Fig. 7e). IL-4 was only detected in splenocyte cultures stimulated with Concanavalin A (data not shown).

Images taken from the bladders showed reduced bladder sizes in those obtained from mycobacteria-treated mice compared with control mice (Fig. 7b). Histological analyses of the bladders showed that the OO-emulsion did not affect the epithelium of the mouse bladder, and no changes were observed compared with the PBS-treated group. Non-mycobacteria-treated animals showed a solid mass inside the bladder lumen, with several hemorrhagic and necrotic foci in the tumor (Fig. 7b). No tumors were present in mycobacteria-treated bladders. Instead, chronic inflammation with moderate to intensive lymphocytic infiltration into the lamina propria and activation of the lymphoid follicles were present (Fig. 7b).

## Discussion

Mycobacteria are peculiar prokaryotes that are characterized by a highly hydrophobic lipid-rich cell wall. This high hydrophobicity is reflected in their tendency to bind to other hydrophobic compounds (Fig. 2a) and to form aggregates when they are in a hydrophilic environment. This characteristic means that it is extremely difficult to generate aqueous preparations of mycobacteria^10^,^21^. Such aggregation could greatly interfere in the interaction between host cells and mycobacteria and, consequently, influence their antitumor efficacy. This drawback is especially important when mycobacteria are used for clinical purposes^11^,^12^.

When manipulating mycobacteria for infection studies, different strategies to disrupt the clumps are conducted (i.e., the use of Tween in liquid cultures, glass/metal ball beating, sonication, syringe passes throughout narrow needles, among others). However, these methods interfere with mycobacteria viability and/or the loss of antigenic molecules from the cell surface^22^. This point is especially significant for BC treatment because the best antitumor action of mycobacteria takes place when live mycobacteria are used^8^,^23^,^24^,^25^. Thus, equilibrium between the disaggregation of clumps and maintaining mycobacteria viability is desired. Moreover, as is widely known, emulsified heat-killed mycobacteria are a potent stimulator of cell-mediated and humoral immune responses^15^. The use of emulsions for heat-killed mycobacteria or cell wall fractions enables the use of these hydrophobic compounds to stimulate the immune system^21^,^26^. Due to this crucial relevance in the medical protocols, many different oil emulsions have been used for the immunotherapy of cancer (Supplementary Table 1). However, emulsions for intravesical live mycobacteria delivery have not been explored until the present work.

Our first relevant result in this work was that among all the mycobacteria emulsions produced, only MO- and OO-in-water emulsions efficiently disaggregated the mycobacteria clumps while maintaining mycobacteria viability. MO is the main oil that is used to emulsify mycobacteria cell walls (Supplementary Table 1) and is currently used for intravesical treatment of BC^26^; however, when the antitumor response *in vitro* was evaluated, MO provided lower inhibition growth proliferation compared with OO-emulsified mycobacteria (Fig. 4). Moreover, OO-emulsified mycobacteria triggered the highest production of cytokines in infected tumor cells among all the infection conditions assayed (Fig. 4). These cytokines have a partial role in the *in vitro* inhibition of cell proliferation^18^ and, notably, have been related *in vivo* to the induction of the immune cascade of events inside the bladder that leads to the elimination of the tumor^23^.

Different hypotheses can be formulated to explain the high efficiency of OO-emulsified mycobacteria to trigger an immune response at this first step. The first idea that arises is that OO-emulsified mycobacteria could be internalized more efficiently than MO and non-emulsified mycobacteria. The internalization of BCG is required for IL-6 release by BC cells^23^,^27^,^28^. However, preliminary results obtained in our laboratory indicated that OO-emulsified mycobacteria are successfully internalized inside T24 cells, but to a similar degree to non-emulsified mycobacteria (manuscript in preparation), which thus discards the first hypothesis. Second, the mechanism of entrance could be different for each emulsion, activating different pathways. The release of the mycobacteria inside the cells and/or the presentation of critical antigens could be improved in OO emulsions. Further studies are then needed to elucidate the mechanism of mycobacteria uptake by using the different vehicles. Finally, we cannot totally discard a synergistic mechanism with the delivery vehicle. Oils have long been used as adjuvants, and they are critical for antigen delivery and the interaction of the immunomodulatory component/s.

A detailed study of the OO/W emulsion illustrated the affinity of live *M. brumae* for OO. As early as in 1931, Reed and Rice described the attraction of live mycobacteria for OO in oil-water systems^29^. Moreover, mycobacteria possess, as explained earlier, a broad spectrum of lipid and glycolipids in their cell walls, which retain oil droplets and stabilize oil-in-water emulsions^30^. The physicochemical characteristics of OO-emulsified *M. brumae* were also analyzed. Our results showed that OO-emulsified mycobacteria present a lower pH (approximately pH 5) and hydrophobicity compared with non-emulsified mycobacteria and, although the zeta-potential values were still negative, those were closer to neutrality when *M. brumae* was emulsified (Fig. 6). Intravesical delivery drugs should possess pH values of approximately 5, low hydrophobicity, and positive zeta-potential values, to facilitate the interaction with the urothelium^31^,^32^. These criteria arise because both the bladder luminal side and mycobacteria are highly negatively charged in terms of the zeta potential, which results in a low probability of irreversible adherence mediated by fibronectin and integrin receptors^3^,^31^. Moreover, the mucin layer is hydrophilic, whereas the mycobacteria are hydrophobic. Therefore, all three parameters were modified in the emulsified versus non-emulsified mycobacteria (Fig. 6), which appears to favor interactions with the urothelium^31^,^32^,^33^. Finally, binding to fibronectin, described by several authors as being crucial to an efficient treatment^34^,^35^,^36^, was increased when mycobacteria were in the emulsified form, indicating another advantageous character for efficacious attachment.

Next, the *in vivo* effect of OO-emulsified mycobacteria in an orthotopic syngeneic mouse model was studied. Remarkably, our results showed that 100% tumor-bearing mice survived when treated with OO-*M. brumae*. The high survival rates that were already obtained using non-emulsified *M. brumae* make it difficult to obtain significant differences between the emulsified and non-emulsified *M. brumae* treated groups without an elevated increase in the number of animals per group, although this option is difficult to justify for ethical reasons. Nevertheless, the high survival rates obtained using OO-*M. brumae* (100%) have never been achieved before with BCG treatment in this animal model^9^,^20^. When the systemic immune response triggered by emulsified-mycobacteria treated mice was studied, the results showed a significant increase of IFN-γ production in spleen cultures (Fig. 7e). The fact that splenocytes specifically restimulated by mycobacteria produce IFN-γ means that memory cells are found in the spleen^37^, driving the shift to a Th1 response that is needed for an effective treatment^25^,^38^. Our results lead us to hypothesize that due to the increased immune response triggered by the instillation of emulsified mycobacteria, could enable modifications in the schedule of instillation, providing improvements for patients: low number of instillations, reduced dose of bacteria per instillation, more spaced instillations or even the duration of the treatment^25^,^39^,^40^,^41^,^42^. Overall, by administrating OO-emulsified mycobacteria it would be possible to find the appropriate pattern of instillations to improve the ratio between therapeutic efficacy and adverse events for the patients, further studies are needed to confirm that point. In fact, the anticancer effects of BCG are regulated by complex immune-related mechanisms^43^. Different authors have described that an insufficient immune response or, by contrast, an overactive response are related to inefficient clearance of the tumors^23^. Our current experiments are addressing this point, and the results will provide us with more information about the possible clinical efficacy of this treatment.

Outstandingly, our results also demonstrate that emulsified *M. brumae* is still a non-pathogenic bacterium. Neither *in vitro* (Fig. 5) nor *in vivo* (Fig. 7d) live emulsified *M. brumae* cells persist a long time, which confirms its non-pathogenicity^7^,^44^ and reinforces *M. brumae* as a safer alternative to BCG for further clinical assays.

### Conclusions and future perspectives

In the present study, we demonstrated the improved characteristics of *M. brumae* emulsified with OO over non-emulsified *M. brumae* in terms of disaggregating mycobacteria clumps while preserving mycobacteria viability, favorable physicochemical properties for *in vivo* attraction to the bladder wall, an innocuous characteristic, and the improved immune response both in *in vitro* cultures and *ex vivo* from treated tumor-bearing mice. In conclusion, when formulated in olive oil-in-water, emulsified *M. brumae* improves its immunostimulatory capacity for non-invasive bladder cancer treatment.

## Materials and Methods

### Bacterial strains and cell lines

*M. bovis* BCG Connaught (ATCC 35745) and *M. brumae* (ATCC 51384) were grown on Middlebrook 7H10 agar (Difco Laboratories, Surrey, UK) supplemented with 10% oleic-albumin-dextrose-catalase enrichment medium at 37 °C for 4 and 1 weeks, respectively.

The human transitional carcinoma cell lines T24 and 5637 was kindly provided by the Cancer Cell Line Repository (RTICCC-PRBB) and was authenticated following short tandem repeat profiling in DSMZ (most recently in September 2014). The mouse BC cell line MB49 was a kind gift from Dr. Mangsbo (Uppsala, Sweden). Cell monolayers were maintained as described previously^9^,^18^.

The preparation of media, manipulation of samples for instillation into the mice, and culturing procedures were always conducted in a biosafety cabinet under sterile conditions.

### Preparation of microemulsions

Microemulsions of mycobacteria were prepared following previously described protocols (Supplementary Table 1), with slight modifications. Four different compounds, OO, SO, SE and MO (all purchased from Sigma-Aldrich, Madrid, Spain), and two different sonication methods were used. Thus, in total, 32 different formulations were evaluated (Table 1).

The first sonication protocol that was used consisted of placing 2 mg of mycobacteria dry-weight together with the oil phase and then adding the aqueous phase up to a volume of 2 mL in a conical glass tube, which was subsequently sonicated in a bath sonicator (BANDERLIN electronic, Berlin, Germany) for 5 minutes at 4 °C^45^. The second sonication method was the rod sonication protocol, which was made in a final volume of 4 mL. For preparing the emulsion with the proportions selected (Table 1), 4 mg of mycobacteria (dry-weight) was placed in a sterile conical glass tube (Duran Group, Wertheim/Main, Germany), and 47.92 mL of a sterile mixture of 16.52% v/v Tween 80 (Sigma-Aldrich) and 83.47% v/v compound was added. Then, a sterile glass rod was used to mix them, and 500 μL of 0.85% w/v NaCl (Panreac, Barcelona, Spain) was added and mixed again. The mixture was sonicated in a bath sonicator for 3 minutes at room temperature (RT). The final volume of the emulsion was adjusted, and the aqueous phase depended on the bacterial concentration that was required for each experiment^46^.

### Microbial adhesion to hydrocarbons (MATH)

To measure the affinity of *M. brumae* for each of the compounds used for the preparation of the emulsions, 0.150 mL of the oil/compound or n-hexadecane (VWR, Merck, Barcelona, Spain) was added to 3 mL of *M. brumae* suspension in PBS (A_600_ 0.44–0.62, referred as A_0_). The mixture was vortexed for 1 minute and settled for 10 minutes. Then, the absorbance of the aqueous phase was measured again (A), and the hydrophobicity index was calculated using the following formula: *hydrophobicity index* = [1 − (A/A_0_) × 100]^17^,^47^.

The presence of bacteria in each phase (oil or water) was visualized using three different microscopy techniques (Supplementary Materials and Methods).

### Viability and clumping of formulated mycobacteria

*M. brumae* viability in each emulsion was determined using two methods: colony-forming unit (CFU) counts and confocal microscopy observation of stained live/dead mycobacteria. For the first method, serial dilutions of each emulsion were platted on Middlebrook 7H10 agar, and the colonies were counted after one week of incubation at 37 °C. For the second method, a LIVE/DEAD^®^ BacLight^TM^ Bacterial viability kit (Life Technologies, Eugene, OR, US) was used to stain *M. brumae* in each formulation. Stained mycobacteria were observed using a TCS-SP5 confocal laser scanning microscope (Leica Microsytems, Mannheim, Germany). HCX PL APO lambda blue 63.0 × 1.40 oil UV objective operating at a zoom of 1.8 was used. To determine both the *M. brumae* viability and the clump size (next paragraph), five horizontal (x-z) optical sections (stepsize 1.51 μm) of 20 fields for each condition were captured. Digital images were processed with Metamorph software (Molecular Devices LLC, Sunnyvale, CA, US) to calculate the percentage of live and dead *M. brumae* cells.

Masks were applied to the stacks, and the area of the *M. brumae* clumps formed in each emulsion were analyzed from the images taken using a confocal microscope, as described above, and were automatically quantified using ImageJ software (National Institutes of Health, Bethesda, MD, US). Areas from 0.7 to 3 μm^2^ were considered to be a single bacterium. Larger areas were considered to be clumps: 3–10 μm^2^ were small clumps, and >10 μm^2^ were large clumps.

### Direct growth inhibition experiments and cytokine analysis

T24, MB49 and 5637 bladder tumor cells were infected with emulsified and non-emulsified mycobacteria at a multiplicity of infection (MOI) of 10:1, and 72 hours post-infection, the cell proliferation was analyzed as described previously^9^,^18^. Cytokine production on cell culture supernatant was measured using commercially available enzyme-linked immunosorbent assay (ELISA) kits according to the manufacturer’s instructions (human IL-8 and IL-6 and mouse IL-6 (BD Pharmingen, San Diego, CA, US) and CXCL1/KC (R&D systems, Minneapolis, MN, US)).

### Survival of mycobacteria inside bladder cancer cells

T24, MB49 and 5637 BC cells (6 × 10^4^ cells/well) were seeded in 48-well tissue culture plates and infected with emulsified or non-emulsified mycobacteria (MOI 10:1). At different time-points after infection, the supernatants were discarded, and the BC cells were lysed^9^. Serial dilutions of cell lysates were plated on 7H10 plates for CFU counting, as described above, or in a 96-well plate 7H10-based spot culture growth assay^48^.

### pH and zeta potential of the microemulsions

pH and zeta potential values were determined in OO-emulsified and non-emulsified mycobacteria using pH strips and a laser-scattering method (Zetasizer Nano ZS, Malvern Instruments, Worcestershire, UK), respectively^32^.

### Adhesion to polystyrene microplate

Fifty microliters of OO-emulsified or non-emulsified mycobacteria were added into BSA-coated (700 μg/cm^2^; Millipore-Kankakee, Il, US), fibronectin-coated (1 μg/cm^2^; Sigma) or non-coated 96-well flat-bottom polystyrene plates in triplicate (Nunc, Roskilde, Denmark). After incubating for 24 hours at RT, five washes with PBS were performed. Adhered mycobacteria were stained with 0.05 mL of crystal violet (Laboratorios Conda, Madrid, Spain) (1%) for 15 minutes at RT, followed by 5 final washes. The absorbance was measured at A_550_ using an ELISA plate reader. As a negative control, 0.05 mL of PBS was used^17^.

### Visualization of the OO/water emulsion

To confirm the O/W nature of the mycobacteria emulsion, the drop test (Supplementary Materials and Methods) and two different microscopy techniques were performed, as described previously^15^. For the light and fluorescent microscopy observation, 2% trypan blue was added to the aqueous phase, and the mycobacteria were stained with Syto^®^ 9 (Life Technologies) when performing the emulsion in OO. Five microliters of the emulsion was placed on a slide, and the coverslip was sealed with transparent nail polish. The samples were observed at 1000X using light and fluorescent microscopes, as described in the Supplementary Material and Methods.

To observe the ultrastructure of the emulsified mycobacteria in a near-native stage, a Field Emission Scanning Electron Microscope (FESEM) was used. Emulsified and non-emulsified *M. brumae* were fixed with 1:1 osmium tetraoxide (4%, TAAB Lab., West Berkshire, UK) at 4 °C for 30 minutes. Then, 5 μL samples were deposited in silicon wafers (Ted Pella, Redding, CA, US) over a period of 1 minute, and excess sample was blotted with Whatman paper, air dried and observed without coating using a FESEM Zeiss Merlin (Oberkochen, Germany) that was equipped with a high-resolution *in-lens* secondary electron detector and operated at 0.8 kV.

### Orthotopic model of bladder cancer and intravesical treatment

Animal experiments were approved by the Animal Care Committee at the Autonomous University of Barcelona, and were carried out in accordance with the approved guidelines. The orthotopic murine model of BC was developed as described previously^9^,^20^,^42^,^49^. Briefly, a tumor was induced in C57Bl/6 female mice (6–8 weeks old; Charles River Laboratories, Barcelona, Spain) by intravesically instilling L-poly-lysine (Sigma-Aldrich) followed by 10^5^ MB49 cells. Mice were then treated intravesically with mycobacteria (2 × 10^6^ BCG or 2 × 10^7^ *M. brumae* cells) in PBS or in OO/W emulsion (schedule shown in Fig. 7a). Control mice were treated with PBS or emulsion without the mycobacteria. The animals were evaluated on a daily basis for their general aspect and behavior and were euthanized when required to avoid unnecessary suffering. After 60 days, the surviving animals were sacrificed. The bladders were collected and pictures taken; the bladders were then fixed and stained for histopathological examination^9^. The spleens were collected, disrupted, serially diluted in PBS, and plated on 7H10 plates for CFU counting.

### Splenocyte cultures

In a second set of experiments (Fig. 7a), mice were sacrificed 29 days after tumor induction, spleens were aseptically removed as explained before, and splenocyte cultures were conducted as previously described^50^. Triplicate wells were stimulated with 1 mg/mL of heat-killed BCG or *M. brumae* cells, or 5 μg/mL of Concanavalin A (ConA, Sigma) as a positive control. After incubation for 72 hours at 37 °C in 5% CO_2_, pooled supernatants from triplicate wells were collected, centrifuged and frozen until use. Supernatants were analyzed for the presence of IFN-γ and IL-4 using commercially available ELISA kits (Mabtech AB, Nacka Strand, Sweden).

### Statistical analyses

The temporal differences in intracellular CFU in *in vitro* cell cultures were analyzed with the non-parametric randomized block analysis of variance for repeated measures Friedman test. For multiple comparisons, data were analyzed using the non-parametric one-way analysis of variance by ranks Kruskal-Wallis H test. Then, pairwise comparisons were performed using the non-parametric Mann-Whitney U test. For comparisons between two groups, data were analyzed using the non-parametric Mann-Whitney U test. For all sequential tests, p values were corrected by the Bonferroni adjustment^51^. For statistical purposes, the values below the detection limit were assigned as half of this limit. All these statistical procedures were performed using SPSS package version 15.0 for Windows (Chicago, IL, US). Log-Rank (Mantel-Cox) tests determined the statistical significance of the Kaplan-Meier survival curves in Prism software version 6.01 for windows (GraphPad Prism, La Jolla, CA, US). Differences were considered to be statistically significant when p < 0.05.

## Additional Information

**How to cite this article**: Noguera-Ortega, E. *et al*. Mycobacteria emulsified in olive oil-in-water trigger a robust immune response in bladder cancer treatment. *Sci. Rep.* **6**, 27232; doi: 10.1038/srep27232 (2016).

## Supplementary Material

Supplementary Material

Supplementary Figure 1

Supplementary Video

## Figures and Tables

**Table 1:** Percentage of hydrophilic and hydrophobic phases and percentage of components in the different emulsions used in the literature (Supplementary Table 1) and in this work.

Phase	Component	Water-in-oil				Oil-in-water					
		Wu		Hwang		Yarkoni		Morales		This work	
Polar		6		8		99		98		99	
	Deionized water		6		8		99		98		99
	Tween 80						0.2		0.50		
	“PBS salts”								0.94		
	NaCl						0.84				0.84
Apolar		94		92		1		2		1	
	Oil*		46.0		50.0		1		2		1
	Tween 80		18.0								0.2
	Span 80		30.0		30.0						
	Brij 98				12.0						

*the experiments were performed using the four different compounds: olive oil (OO), soybean oil (SO), squalene (SE), and mineral oil (MO). Phosphate buffered saline (PBS).
