# Supplementary material for: Mycobacteria emulsified in olive oil-in-water trigger a robust immune response in bladder cancer treatment
Source: Sci Rep. 2016 Jun 6;6:27232. doi: 10.1038/srep27232 (PMC4893706; doi:10.1038/srep27232)
Supplement: Supplementary Material [file srep27232-s1.doc]

**Mycobacteria emulsified in olive oil-in-water trigger a robust immune response in bladder cancer treatment**

**Estela Noguera-Ortega^1^, Núria Blanco-Cabra^1^, Rosa Maria Rabanal^2^, Alejandro Sánchez-Chardi^3^, Mónica Roldán^3^, Sandra Guallar-Garrido^1^, Eduard Torrents^4^, Marina Luquin^1^, and Esther Julián^1^,***

^1^Departament de Genètica i de Microbiologia, Facultat de Biociències, Universitat Autònoma de Barcelona.

^2^Unitat de Patologia Murina i Comparada, Departament de Medicina i Cirurgia Animals, Facultat de Veterinària, Universitat Autònoma de Barcelona.

^3^Servei de Microscopia, Universitat Autònoma de Barcelona

^4^Bacterial Infections and Antimicrobial Therapy group, Institute for Bioengineering of Catalonia (IBEC).

***** esther.julian@uab.cat

**Supplementary Table 1.** Review of formulations of mycobacteria compounds for treating different cancer types.

| Mycobacteria/drug | Emulsion | Oil | Cancer treatment | Reference |
| --- | --- | --- | --- | --- |
| BCG cell wall skeleton | O/W | mineral oil | Guinea pig hepatoma | 1 |
| M. kansasii and M. smegmatis * cell walls | O/W | mineral oil | Guinea pig hepatoma | 2 |
| BCG cell walls and heat killed M. smegmatis | O/W | Drakeol 6VR (mineral oil) | Guinea pig hepatoma | 3 |
| M. smegmatis Cord factor | O/W | peanut oil | Murine L1210 leukemia | 4 |
| Killed BCG, M. phlei, and M. smegmatis | O/W | Squalene | Murine fibrosarcoma | 5 |
| M. phlei cell wall extract plus DNA | O/W | mineral oil | Rat prostate and murine bladder cancer | 6 |
| BCG cell wall skelleton | O/W | squalane or mineral oil | Murine lung metastasis from colon carcinoma and melanoma | 7 |
| BCG cell wall skelleton | O/W | Drakeol (mineral oil) | Murine melanoma | 8 |
| BCG cell wall skeleton | O/W | Squalane | Murine Lewis lung carcinoma | 9 |
| Mitomycin-C | O/W | soybean oil | Human lung cancer adenocarcinoma and epidermoid carcinoma ** | 10 |
| Cysplatin | W/O | soybean oil | Bladder cancer ** | 11 |
| Plasmid DNA *** | W/O | olive oil | Murine skin delivery | 12 |
| BCG Glycolipid A1 | - | olive oil | Guinea pig hepatoma | 13 |

Review of W/O and O/W formulations of mycobacteria compounds or drugs for treating different types of cancer. * It was wrongly classified as M. phlei ^5^,** in vitro studies, *** plasmid encoding chloramphenicol acetyltransferase or human INF-α2.

**Supplementary Materials and Methods.**

**Microbial adhesion to hydrocarbons (MATH)**

To corroborate the results that were obtained by the absorbance measurements, the affinity of *M. brumae* for each compound was also studied by microscopy. Thus, 20 µL of the interphase, prepared as explained in the materials and methods section of the main manuscript, were visualized by phase contrast microscopy (Axio Imager.M2, Zeiss, Oberkochen, Germany) to verify the presence of the bacteria in each phase. In another set of experiments, 2% trypan blue (Sigma-Aldrich, Madrid, Spain) was added to the aqueous phase, and the mycobacteria were stained with Syto® 9 (Life Technologies, Eugene, OR, US); the interface was then observed by bright-field and fluorescent microscopy DM6000 B, and the images were captured using a digital camera DFC480 (both from Leica microsytems).

**Droplet test**

To confirm the O/W nature of the emulsion developed, the ”droplet test” was performed ^14^. Twenty microliters of the trypan blue-stained emulsion were dropped in the bottom half of a polystyrene Petri dish filled with 20 mL of cold distilled water. The behavior of the drops was recorded with a photo camera (Nikon). Based on the literature, a W/O emulsion was designed ^15^ to compare the behavior of the O/W emulsion that was made in our laboratory. Briefly, 47.92 μL 0.85% w/v NaCl was mixed with *M. brumae*, and then 500 μL of a sterile mixture of 16.52% v/v Span 80 in olive oil was added to the mixture and sonicated.

**Supplementary Figures.**


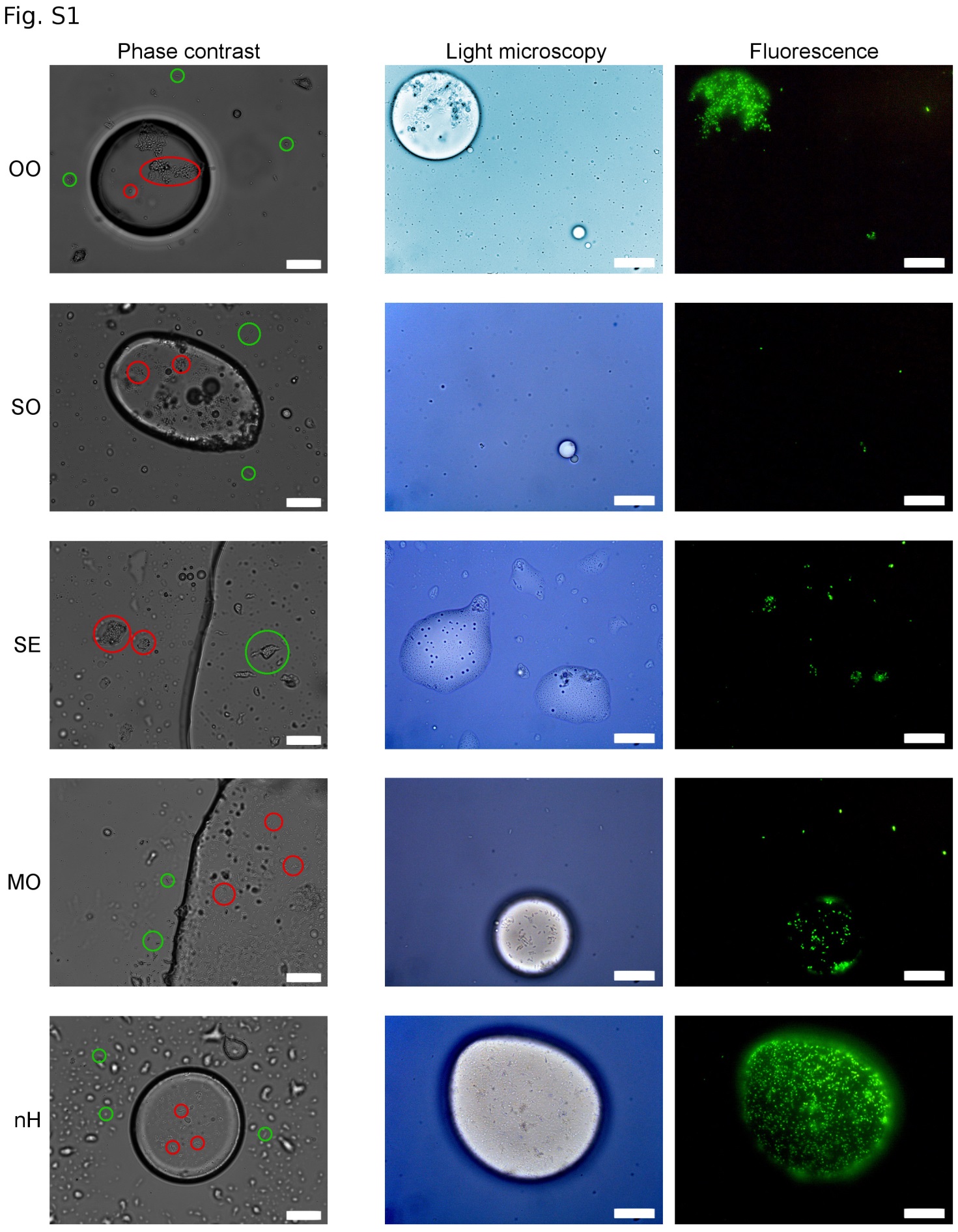


**Supplementary Figure 1.** Detail of *M. brumae* in the oil-water interface of the MATH assay. In the first column representative images of the oil-water interface from the MATH assay using the different compounds are shown. Red and green circles indicate *M. brumae* in the oil and water phases respectively. In the second and third column representative images of the MATH assay are shown: the aqueous phase (seen in light field images) was stained with Trypan blue, and the *M. brumae* cells (seen in fluorescence images) with Syto® 9. OO, olive-oil; SO, soybean oil; SE, squalene; MO, mineral oil; nH, n-hexadecane. Scale bar, 20 μm.

**Supplementary Figure 2.**

Video corresponding to the behaviour in cold water of *M. brumae* formulated (left) in OO emulsion or non-formulated (right).

**References**

1. Meyer, T. J., Ribi, E. E., Azuma, I. & Zbar, B. Biologically active components from mycobacterial cell walls. II. Suppression and regression of strain-2 guinea pig hepatoma. *J. Natl. Cancer Inst.* **52,** 103–11 (1974).

2. Gray, G. R. *et al.* Immunotherapy of cancer: tumor suppression and regression by cell walls of Mycobacterium phlei attached to oil droplets. *J. Natl. Cancer Inst.* **55,** 727–30 (1975).

3. Yarkoni, E., Rapp, H. J. & Zbar, B. Immunotherapy of a guinea pig hepatoma with ultrasonically prepared mycobacterial vaccines. *Cancer Immunol. Immunother.* **2,** 143–146 (1977).

4. Leclerc, C. *et al.* Nonspecific immunoprevention of L1210 leukemia by cord factor (6-6? dimycolate of trehalose) administered in a metabolizable oil. *Cancer Immunol. Immunother.* **1,** (1976).

5. Yarkoni, E. & Rapp, H. J. Immunotherapy of experimental cancer by intralesional injection of emulsified nonliving mycobacteria: comparison of Mycobacterium bovis (BCG), Mycobacterium phlei, and Mycobacterium smegmatis. *Infect. Immun.* **28,** 887–92 (1980).

6. Morales, A., Chin, J. L. & Ramsey, E. W. Mycobacterial cell wall extract for treatment of carcinoma in situ of the bladder. *J. Urol.* **166,** 1633–8 (2001).

7. Yoo, Y. C., Hata, K., Lee, K. B. & Azuma, I. Inhibitory effect of BCG cell-wall skeletons (BCG-CWS) emulsified in squalane on tumor growth and metastasis in mice. *Arch. Pharm. Res.* **25,** 522–7 (2002).

8. Akazawa, T. *et al.* Adjuvant-Mediated Tumor Regression and Tumor-Specific Cytotoxic Response Are Impaired in MyD88-Deficient Mice. *Cancer Res.* **64,** 757–764 (2004).

9. Murata, M. Activation of Toll-like receptor 2 by a novel preparation of cell wall skeleton from Mycobacterium bovis BCG Tokyo (SMP-105) sufficiently enhances immune responses against tumors. *Cancer Sci.* **99,** 1435–40 (2008).

10. Kotmakchiev, M., Kantarcı, G., Çetintaş, V. B. & Ertan, G. Cytotoxicity of a Novel Oil/Water Microemulsion System Loaded with Mitomycin-C in In Vitro Lung Cancer Models. *Drug Dev. Res.* **73,** 185–195 (2012).

11. Hwang, T.-L., Fang, C.-L., Chen, C.-H. & Fang, J.-Y. Permeation enhancer-containing water-in-oil nanoemulsions as carriers for intravesical cisplatin delivery. *Pharm. Res.* **26,** 2314–23 (2009).

12. Wu, H., Ramachandran, C., Weiner, N. D. & Roessler, B. J. Topical transport of hydrophilic compounds using water-in-oil nanoemulsions. *Int. J. Pharm.* **220,** 63–75 (2001).

13. Reggiardo, Z. Antitumor activity of mycobacterial glycolipid A1. *Infect. Immun.* **21,** 914–7 (1978).

14. Lindblad, E. B. in *Vaccine Adjuvants: Preparation Methods and Research Protocols* (ed. O’Hagan, D.) 49–63 (Humana press, 2000).

15. *The HLB System: a time-saving guide to emulsidier selection*. (ICI Americas Inc., 1980). at <http://www.ncbi.nlm.nih.gov/pubmed/21924194>
