# Supplementary figures and images for: Mycobacteria emulsified in olive oil-in-water trigger a robust immune response in bladder cancer treatment
Source: Sci Rep. 2016 Jun 6;6:27232. doi: 10.1038/srep27232 (PMC4893706; doi:10.1038/srep27232)

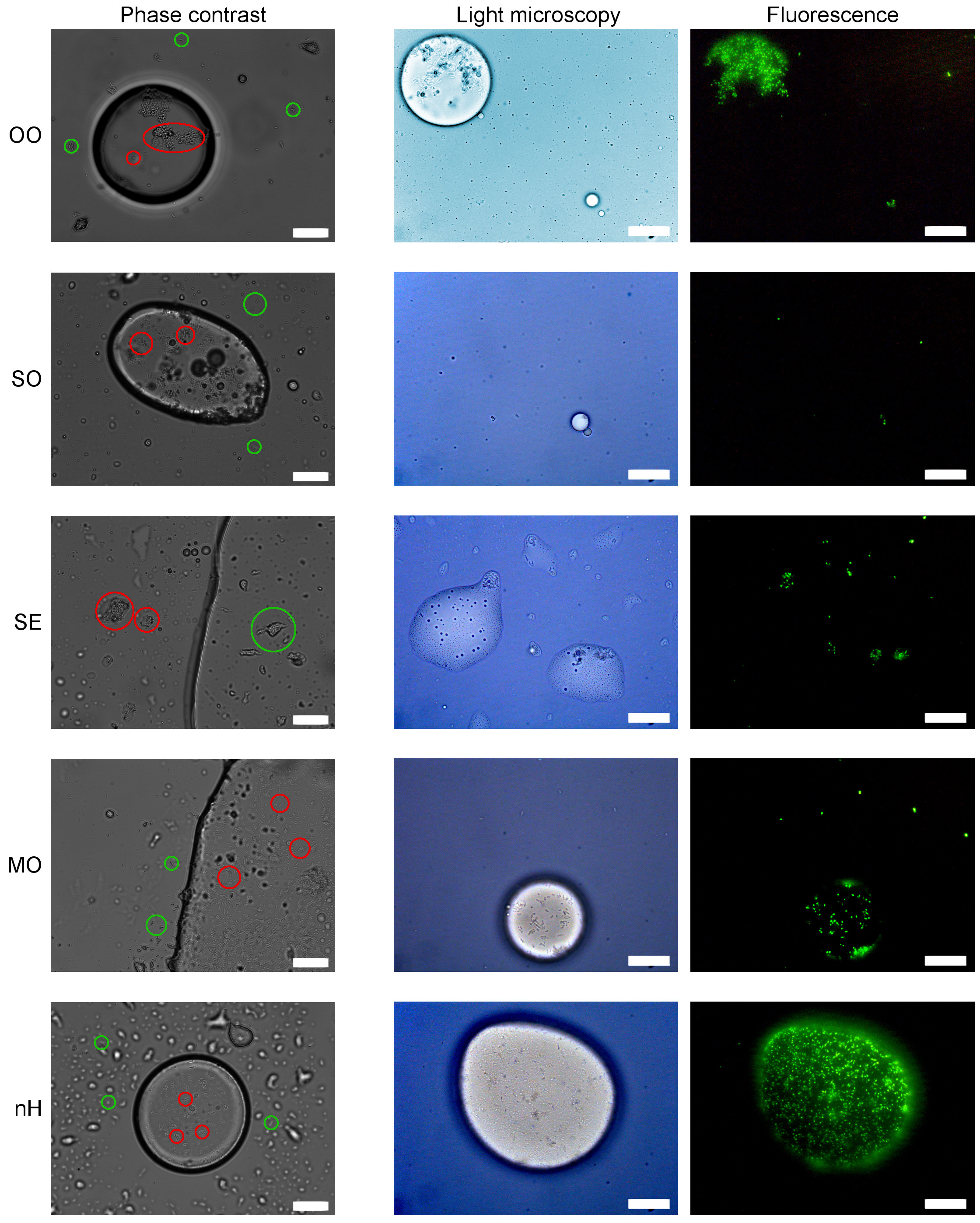

Supplement: Supplementary Figure 1 [file srep27232-s2.jpg]
